# Opaque Optic Nerve Fibres in the Retina

**Published:** 1873-05

**Authors:** A. W. Calhoun

**Affiliations:** Georgia


### FOREIGN CORRESPONDENCE.

---

### OPAQUE OPTIC NERVE FIBRES IN THE RETINA.

BY A. W. CALHOUN, M.D., OF GEORGIA.

Under this title is comprised an anomaly of the retina, which is not of an every-day occurrence even in the practice of the busiest ophthalmologists; still it has often enough been observed and sufficiently accurately pictured by different oculists busying themselves specially with the ophthalmoscope, as possibly not to warrant any further discussion or description upon the simple ground of there being nothing new to be communicated in reference to it. Nevertheless, I venture a few words additional to what may have been previously written, hoping to give some if not entirely new, at least interesting facts, which circumstance may, in this instance, suffice as an extenuation of what I may have to remark; particularly since our memory can not be too often impressed or refreshed with these anatomical peculiarities of the interior of the eye, a knowledge of which is absolutely essential to guard us against a false diagnosis in regard to certain intraocular affections, which in outward appearance, form, etc., strikingly resemble the above anomaly, and which require considerable practice and close scrutiny, by means of the ophthalmoscope to be in condition to clearly distinguish the one from the other.

To properly comprehend the abnormal condition, we must first understand the normal; to do which we take a vertical cut through the optic nerve of a normal eye for microscopical examination, and observe what there presents itself for study. In general, an ordinary primitive nerve fibre, from whatever part of the body, consists of a more or less cylindrical thread, composed of a sheath externally, whose contour is strongly marked, and the medullary substance internally. Such a fibre is untransparent, but where the medullary substance loses the surrounding sheath, from that point the fibre becomes more or quite transparent, to continue in the form of what is known as the axis cylinder. In the optic nerve, as the bundles of fibres approach the lamina cribrosa, each fibre is seen to possess its necessary sheath, and the interior opaque medulla. The medullary sheath ceases in each fibre at this point, (lamina cribrosa) and the clear, transparent medulla or its axis cylinder passes through as a bundle or single fibre, to become lost in the retina itself. If the cut is fine enough, not only can the exact termination of the membranous sheath in each fibre be distinctly traced, but also the contained substance or marrow—now to great extent transparent after being relieved of its covering in its passage through the microscopically small orifices of the cribriform plate or lamina cribrosa of the sclera—can be followed for a short distance, as each turn in different directions from the edge of the optic disc, until they become blended with the other elements of the retina, whose appearace is too well known to require a further repetition.

After this short explanation of the condition of the optic nerve fibres approaching and within the normal eye, it is easily understood what the nature of the anomaly in consideration is, which anatomically considered, is as follows:

Instead of each fibre losing its sheath at the lamina cribrosa, as is the case in every eye with a few rare exceptions, the latter maintains its seat around the body of a greater or less number of fibres passing into the retina in the same state that characterized them when composing the body of the nerve posterior to the bulbus. These fibres extend variable distances into the retina, abruptly losing this peculiar character or state, and pass over into the true or normal transparent nervous constituent of the retina. The above is observed when certain fibres continue uninterruptedly from the body of the nerve to the interior of the eye, possessing unbroken the same peculiarity in each location; but still more remarkable is it when a fibre loses its medullary cover at the lamina cribrosa, proceeding for a certain distance in its transparent normal shape, and again at some point more or less remote from the disc, resuming its opaque appearance—standing forth as an island, as it were, and communicating with the shaft or body of the optic nerve through its medullary substance.

So much as explanatory of the direct cause of the anomaly, and a few words as to its appearance when examined with the opthalmoscope, giving a very impressive picture, but which can readily be confusing to the unpracticed, since certain diseases of the retina and neighboring structures offer at first sight a deceptive similarity.

In most cases, commencing upon the edge of the entrance of the optic nerve, or rather as a continuation of the same on one or more sides, is seen a peculiar white spot, flame-like in general aspect, of an irregular form, distinctly and exactly circumscribed and presenting a characteristic bright pearly glance. This often extends some distance into the retina, either singly or corresponding to the same appearance on the opposite side of the nerve, or as occasionally happens, springing out as small, short zig-zag projections from many and different sides of the optic disc at the same time. In one case which I examined a short time since, these opaque, brilliant, white, glancing fibres proceeded in a tolerably broad compact bundle from the upper and lower margins of the nerve, in the direction of the periphery of the retina, and to the extent to which they advanced, dividing the latter into two symmetrical halves, suddenly ceasing in irregularly notched extremities. Also in the face of the disc itself occurs this anomaly, giving a more peculiar view than when seen within the retinal tissue. The papilla or entrance of the optic nerve being naturally of an opaque, slightly blood-tinged white color, shows forth the few fibres still retaining their sheaths with double effect as a small limited spot, glancing still more brightly than when contrasted against the retina as a background. They usually lose this character upon the edge of the nerve, and proceed with the other fibres of the retina in a normal state. What is still more curious, is that not unfrequently the same anomaly occurs in some portion of the retina quite disconnected with and some distance from the nerve disc; possessing the same characters as those occurring in the different situations just mentioned; standing forth more prominently with well marked outlines and a clearer glance, from being hemmed in on all sides by the normal retina, the transition from the one into the other being abrupt and conspicuous. In an occasional case these collections of fibres appear as if raised above the level of the surrounding parts, the retinal vessels reaching its edge and passing underneath, to make their appearance again on the opposite side. Very seldom do the vessels pass over the anterior surface, and while underneath they either seem thickly veiled or disappear altogether. Perhaps these spots or patches are more definitely outlined when examined with a strong light in the direct image; however, they are also very clear when viewed in the indirect.

This remarkable ophthalmoscopic picture presents the greatest similarity to that seen in the eyes of certain quadrupeds—rabbits for intance—where the bright white opaque fibres spread out in broad, flat bundles, to the right and left; and which, in all probability, depend upon the same causes as when occurring in the human subject, viz: that the corresponding nerve fibres have not yet dispensed with their white medullary sheaths or membranes.

Strange, and yet at the same time fortunate, is it that these opaque fibres never extend to the outer side of the papilla towards the macula lutea. No such case has yet been recorded in any of the most extended observations, and but extremely seldom does it occur directly inwards from the nerve. In most of the many cases that I have examined, the flame-like projecting fibres sprang immediately upwards and downwards, intruding occasionally somewhat to the one or the other side. Were any of the nerve fibres opaque which lead to or in the region of the macula lutea, acute vision would necessarily be more or less injuriously affected, according to their proximity to this point; for in and around this spot are gathered the most sensitive elements of the retina, and hence is here seated, so to speak, the centre of that acute and exact sight that we are continually called upon to exercise each day of our lives.

According to Prof. Jaeger this anomaly is nothing more than an enlarged optical disc, and in a certain sense this idea is perfectly comprehensible. We all possess what is known as the "blind spot," but it occupies such a comparatively small surface in the interior of the eye, and interfering to no degree with the powers of vision, that we but seldom observe it. But each one of us can observe it however, and in a manner picture it to himself by the following simple experiment: Take for instance any object or spot on a wall, or better, a chalk mark upon a black-board, and steadily and firmly fix the sight of the one eye upon it while the other is held closed either with the hand or by means of a bandage or handkerchief, etc., and then with a pencil, or any substance having a white end, held in the other hand against the wall or board, but off to one side out of the field of vision, slowly approach the spot carefully fixed with the opaque eye. As soon, of course, as the pencil comes within the range of vision, we perceive its white head, but as it slowly nears the spot on the board it finally reaches a point where it is no longer seen, but being moved a fraction further again appears. Mark the point at which the head of the pencil begins to disappear, and repeat the same maneuvre from all the different directions—viz., from above and below, from each side, etc., keeping the eye all the while as near as possible immovably fixed upon the spot approached, and we have drawn upon the board a nearly circular figure corresponding to the form of the optic disc or to the so-called "blind spot." Let an individual try this who has these abnormal opaque fibres developed in his eye, and it will be found that the only difference between his eye (excluding other complications) and one normal in all respects, consists simply in the fact that in certain directions the white pencil head reaches earlier in the one than the other, that limit where it ceases to be seen, and that on the board is pictured a more extensive and more irregular figure, corresponding to the blind spot, and hence showing the disc of the optic nerve in an enlarged state.

We can scarcely look upon this as a diseased condition of the eye, for in most instances the individuals are in no way whatever troubled by its presence. Frequently, however, it is accompanied by some anomaly of refraction, such as a high degree of hypermetropia or myopia, which of course impose upon their victims well known and characteristic optical disturbances. The complication by either of these is simply a coincidence, not the least connection existing between the one and the other.

In the case of a boy, aged fourteen years, who was brought into the ophthalmoscopic class in the clinic of Prof. Arlt, for the purpose of demonstrating a normal retina, it was found that the opaque fibres were very extensive in one eye, the other being unaffected and quite normal. He had never discovered any difference in vision between the two eyes, and in fact a minute examination would have only shown the blind spot in this, the abnormal eye, occupying a larger space than in the other.

Another and well-spoken case was that of a girl, aged seventeen, who came into the clinic of Prof. Jaeger, to accompany a younger sister who was under treatment for some internal disease of the eye, and through curiosity or for the sake of practice, her eyes (the older sister's) were ophthalmoscopically examined, and much to her surprise and fright, as well as to the joy of the examiner—flattering his diagnostic powers—large patches or bundles of opaque fibres were found. Had not this accidental examination proven to the girl that a very seldom anomaly lay cooped up in the interior of her bulbus, no other circumstance, such as defective vision depending upon the opacity of these fibres, would probably have ever acquainted her with the fact.

I have seen no case where it existed upon both eyes, and if I am not mistaken, but few instances are recorded of its being other than a one-sided peculiarity. It is, of course, to be found in all ages; quite young children, as well as aged persons, being in the same manner affected, since, without doubt, the anomaly is congenital. The fact that we have occasion less frequently to examine the eyes of children than adults for different troubles, and that their vision is in no way, or but in the very slighest degree, affected by the presence of this anomaly, accounts for its apparent extreme rarity in the early years of life. In most of those cases where it has been seen in children, some other disturbing anomaly, as of refraction, existed; and in examining the latter, the other was also brought to light. Only extremely seldom is this abnormity to such an extent developed as of itself to injuriously interfere with a normally sharp, acute perception of all objects.

The diseases with which it is most likely to be confused, are certain forms of choroiditis and retinal exudations, but practice with the ophthalmoscope and a particular careful investigation on the part of the observer, will, in most cases, lead him to a correct diagnosis.

Of the greatest diagnostic importance in reference to both of these affections, but specially the former, is the want of any defect of vision in connection with the anomaly itself; while on the other hand choroiditis is, without exception, followed by a greater or less degree of defective sight, depending upon the locality and the extent of the inflammatory and atrophic change undergone by the choroidea. Corresponding to the white atrophic spots resulting from the choroiditis, the choroidal vessels are seen passing from one edge to the other, and generally themselves presenting an appearance of partial atrophy, considerably diminished in size and running in a straight course from one point to the other, the walls of each having lost to a certain extent their characteristic bright red color; whereas in the normal choroidea the vessels run in a more or less tortuous, serpentine direction. The atrophic spot is not closely circumscribed, but gradually goes over into the sound choroidea, by degrees assuming its natural color, and other characters. Occasionally, also, in these spots the vessels completely disappear, becoming entirely atrophied according to the extent of atrophy of the corresponding portion of choroidea. This refers only to that form of choroiditis ending in the above mentioned atrophy of a greater or less circuit of the choroidal tissue and the complete loss of its pigment; other forms also frequently occurring, but are in no way to be confounded with the anomaly in question.

The other affection from which we can find difficulty in distinguishing the anomaly is retinal exudation, but even such a difficulty is more apparent than real. The exudations as a result of retinitis can, as may be supposed, take place in any part of the retina, and usually they sit near or upon some one or more of the retinal vessels. They present a cloudy, white appearance, sometimes raised a little above the level of the adjacent retina, and when resting upon any of the vessels either hide them thoroughly or cover them as with a thick veil, allowing them to show themselves at different intervals in their passage underneath the exudation. The immediate vicinity of the exudation partakes also of the cloudiness, it being not easy to set a limit to its extent, or to say where it begins and where it ends, the haziness so gradually fading into the natural and healthy retina. If the retinitis has reached any high degree, and more especially if the exudations sit near the papilla, the latter is also affected, jutting forward under the influence of the swelling of the fibres of the nerve, and assuming a more reddened hyperæmic, or even a dirty opaque appearance, with an indefinable, indistinct circumference, according to the intensity of the general inflammation and the associate trouble of the nerve itself. In such cases disturbances of vision are necessarily to greater or less extent present.

If the chief characteristics of the opaque nerve fibres are borne in mind—viz., the glistening whiteness, the usual connection with the nerve itself, commencing mostly upon its very edges, the abrupt and zig-zag manner of ceasing, the failure of every inflammatory symptom in the immediate vicinity, such as a dull opacity in the adjoining retina, etc., and above all the total absence of any defect of vision, little difficulty can be experienced in coming to a conclusion.

These two diseases offer, of course many other symptoms, which in other respects are of much importance, but only those are here mentioned that are of particular worth in separating them from this anomaly, in itself no disease, but in many points presenting a marked likeness.

Were we able to do anything against such a state of things in the interior of the eye, common reason would forbid us, since no outward or visible deformity exists, and no dependent visionary disturbance ever calls for our aid, numbers of individuals no doubt passing through a long life, happily ignorant of the existence of any such abnormity, a knowledge of which might be productive of much alarm but never of danger.

VIENNA, AUSTRIA, March 15, 1873.

